# The Guareschi Pyridine Scaffold as a Valuable Platform for the Identification of Selective PI3K Inhibitors

**DOI:** 10.3390/molecules200917275

**Published:** 2015-09-18

**Authors:** Ubaldina Galli, Elisa Ciraolo, Alberto Massarotti, Jean Piero Margaria, Giovanni Sorba, Emilio Hirsch, Gian Cesare Tron

**Affiliations:** 1 Dipartimento di Scienze del Farmaco, Università del Piemonte Orientale “A. Avogadro”, Largo Donegani 2, Novara 28100, Italy; E-Mails: ubaldina.galli@uniupo.it (U.G.); alberto.massarotti@uniupo.it (A.M.); giovanni.sorba@uniupo.it (G.S.); 2 Department of Molecular Biotechnology and Health Sciences, Molecular Biotechnology Center, University of Torino, Via Nizza 52, Torino 10126, Italy; E-Mails: elisa.ciraolo@unito.it (E.C.); jeanpiero.margaria@unito.it (J.P.M.)

**Keywords:** PI3 kinase, pyridine, Guareschi reaction, isoforms, selectivity

## Abstract

A novel series of 4-aryl-3-cyano-2-(3-hydroxyphenyl)-6-morpholino-pyridines have been designed as potential phosphatidylinositol-3-kinase (PI3K) inhibitors. The compounds have been synthesized using the Guareschi reaction to prepare the key 4-aryl-3-cyano-2,6-dihydroxypyridine intermediate. A different selectivity according to the nature of the aryl group has been observed. Compound **9b** is a selective inhibitor against the PI3Kα isoform, maintaining a good inhibitory activity. Docking studies were also performed in order to rationalize its profile of selectivity.

## 1. Introduction

Class I phosphatidylinositol-3-kinases (PI3Ks) [1] are lipid kinases able to phosphorylate the hydroxyl group at the three position of the phosphatidylinositol-4,5-biphosphate (PdtIns(4,5)P_2_). They exert a pivotal role on several cellular processes such as proliferation, survival, motility, metabolism, and differentiation [2].

Class I PI3Ks are usually heterodimers consisting of a catalytic subunit and an adaptor/regulator subunit. All class I catalytic subunits share substantial homology and are referred to as p110 subunits. The contribution of each of the four isoforms of class I PI3Ks has been elucidated in detail, as their tissue localization [3]. For example, in mammals, whereas the expression of PI3Kδ is restricted to the immune system, PI3Kα and PI3Kβ are ubiquitously expressed, and the p110γ catalytic isoform is mainly expressed in leukocytes. For these reasons, the identification of PI3K inhibitors with a different profile of selectivity can be useful for the treatment of different diseases [4].

Over the last decades, pan-PI3K inhibitors [5,6] were extensively investigated for the treatment of cancer; however, the use of isoform-selective PI3K inhibitors seems to be a better strategy in cancer therapy in order to reduce the side effects associated with pan-PI3K inhibitors [7,8]. Alterations in the PI3K pathway, such as PI3Kα mutations, over-expression, or gene amplification, and PTEN (phosphatase and tensin homologue) phosphatase deletion, have been identified in several types of cancer (mammary gland, colon, prostate, brain) [5,6]. Moreover, recent evidence demonstrated that PI3Kα selective inhibitors [7] are more effective against tumors with mutations in the PI3Kα gene. PI3Kβ-selective inhibitors have been proposed in the treatment of PTEN-deficient tumors [9,10,11], while PI3Kδ inhibitors are currently under evaluation in clinical trials for the treatment of some specific lymphoproliferative diseases including chronic lymphocytic leukemia (CLL), multiple myeloma, and Hodgkin’s lymphoma [12,13,14,15]. In this context, a more specific targeted therapy instead of the use of pan-PI3K inhibitors could help to maximize the antitumor effects, reducing the potential side effects (hematological complications, insulin resistance) associated with an indiscriminate inhibition of all the PI3K isoforms.

The identification of selective PI3K inhibitors is still a topic of research both in industry and academia and, to date, several potent PI3K inhibitors, able to compete with the ATP binding site, have been reported [16,17,18,19].

**Figure 1:**
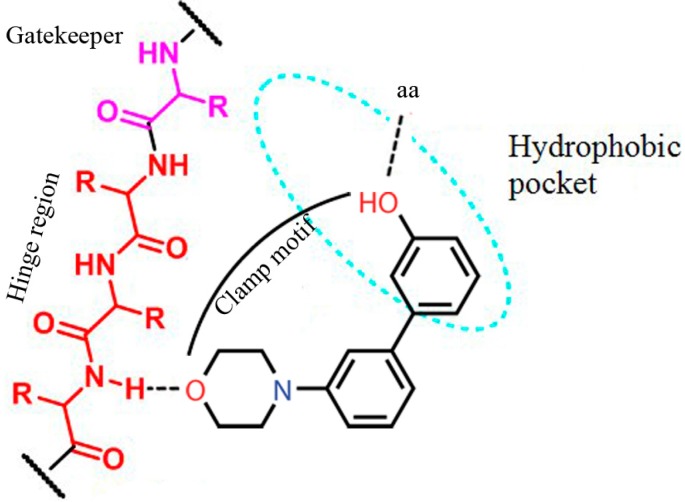
The clamp motif strategy for the identification of novel PI3K inhibitors (in the case of PI3Kγ, the phenol group interacts with Tyr867, while morpholine interacts with Val882).

The intellectual property of ATP-competitive inhibitors is highly over-crowded, rendering the identification of novel molecular entities a daunting task for medicinal chemists. One important feature present in most of the ATP-competitive PI3K inhibitors is the presence of the so-called clamp motif [20] formed by a morpholine, a hinge-binder typical for lipid kinases, and a meta-substituted phenol (or one of its bioisosteres) grafted onto a heteroaromatic ring (Figure 1).

The strategy of the clamp motif grafted to a heteroatomatic ring has been utilized by several research groups. For example, cyanuric chloride was used as the starting material, exploiting the different reactivity of chlorine atoms in the nucleophilic aromatic substitution to add different hinge-binders [21].

**Figure 2:**
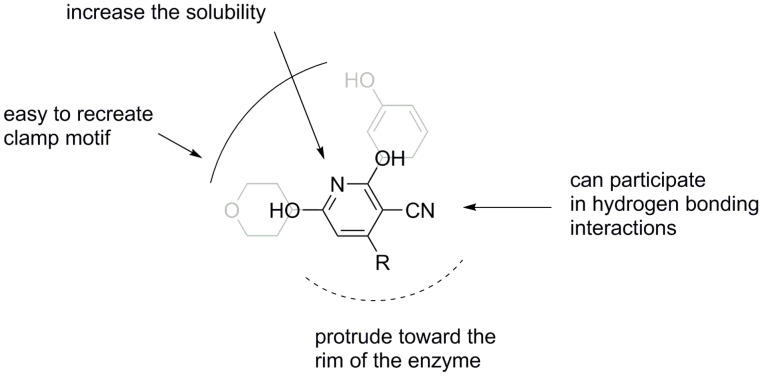
The Guareschi pyridine scaffold can be advantageously used for the design of novel PI3K inhibitors.

With this in mind, looking for novel PI3K inhibitors, we focused our attention on the neglected 3-cyano-2,6-dihydroxypyridine scaffold, which can be obtained via Guareschi reaction [22], as a valuable chemical platform. Indeed, it contains all the chemical functions to build the clamp motif and a group pointing towards the rim of the binding pocket. Furthermore, the pyridine ring can increase the solubility, while the cyano group is an added bonus as it might play an active role in the kinase inhibition [23] or it can be considered as a handle for further chemical manipulations (Figure 2). As far as we know, this is the first attempt to use this chemical scaffold in the kinase inhibitor field.

As the amino acid residues located at the entrance to the catalytic site are not conserved in the different PI3K isoforms [24], we decided to maintain the morpholine and phenol clamp motifs, exploring the C_4_-position of the pyridine ring introducing different aromatic groups. It would be expected that these groups, which protrude outside the ATP-binding pocket, might give additional contacts with the less conserved amino acid residues, altering the profile of selectivity.

The synthesis, the biological activity, and the PI3K subtype selectivity of the compounds synthesized are presented herein.

## 2. Results and Discussion

**Scheme 1:**
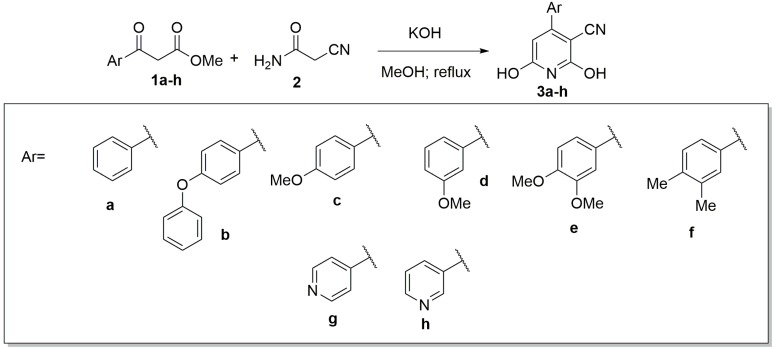
The Guareschi pyridine reaction.

The 4-aryl-3-cyano-2,6-dihydroxypyridines (**3a**–**h**) were synthesized using the improved Guareschi pyridine synthesis reported by Bobbit and Scola [25]. This methodology consists of the condensation of aryl β-keto esters (**1a**–**h**) and cyanoacetamide (**2**) in potassium hydroxide at reflux using methanol as solvent (Scheme 1).

**Scheme 2:**
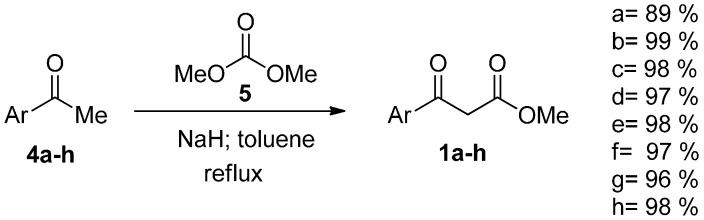
Synthesis of aryl β-keto esters.

Aryl β-keto esters **1a**–**h** were prepared from the corresponding commercially available aryl methyl-ketones **4a**–**h** by deprotonation with sodium hydride and quenching with dimethylcarbonate (**5**) [26] (Scheme 2).

Condensation of these crude dicarbonyl compounds with cyanoacetamide under basic conditions afforded the corresponding 4-aryl-3-cyano-2,6-dihydroxypyridines (**3a**–**h**) (Scheme 1). Despite our effort to optimize this reaction, the compounds were obtained in modest yields (Table 1).

**Table 1:** Yields of 4-aryl-3-cyano-2,6-dihydroxypyridines.

Compound	Yield (%)
**3a**	41
**3b**	15
**3c**	14
**3d**	28
**3e**	9
**3f**	27
**3g**	26
**3h**	10

This result can be ascribed to the behavior of β-ketoesters which decarboxylate at high temperatures, and then they underwent ester hydrolysis to β-ketoacids under these basic conditions. In confirmation of this situation, we recovered aryl methyl-ketones from the reaction mixture.

Unfortunately, under room temperature, the Guareschi reaction did not take place and the use of a milder reported procedure (1,4-diazabicyclo[2.2.2]octane or DABCO^®^, r.t.) failed to afford the desired products [27].

**Scheme 3:**
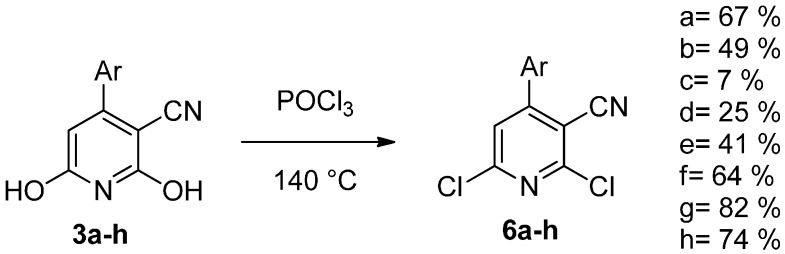
Synthesis of the dichloride analogues.

The key intermediates (**3a**–**h**) were then converted to the corresponding dichlorides (**6a**–**h**) with phosphorus oxychloride (Scheme 3).

**Scheme 4:**
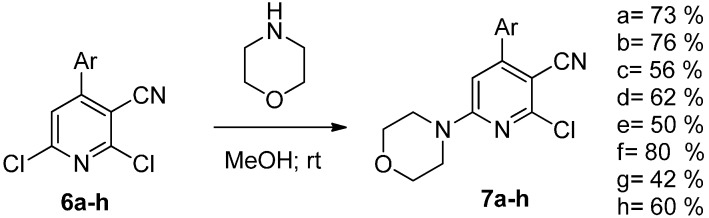
Regioselective addition of morpholine at the six position.

The regioselective reaction with morpholine in methanol afforded the derivatives **7a**–**h** (Scheme 4). A recent paper demonstrated that regioselectivity at the six position can be easily achieved using a polar solvent like methanol [28].

**Scheme 5:**
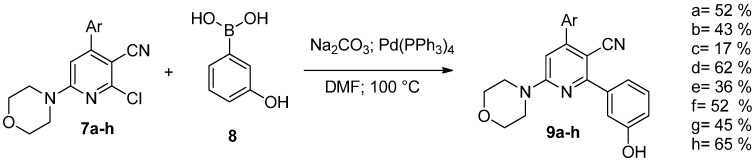
Synthesis of compounds **9a**–**h**.

Finally, the desired tetrasubstituted pyridines **9a**–**h** were obtained via Suzuki coupling with 3-hydroxyphenylboronic acid (**8**) (Scheme 5).

All the synthesized compounds were then tested *in vitro* for their ability to inhibit the kinase activity of the PI3Kα, PI3Kβ, PI3Kγ, and PI3Kδ isoforms in a luminescent kinase assay (ADP-GloTM kinase assay) using purified, recombinant human enzymes and ATP. IC_50_ values were subsequently determined.

As shown in Table 2, the compounds display an activity at low micromolar concentrations against the four isoforms, changing their profile of selectivity according to the type of aryl group on the four position of pyridine. In particular, for this series of compounds, the molecule **9b** stands over all the others, showing a remarkable profile of selectivity toward the PI3Kα isoform.

**Table 2:** IC_50_ inhibitory profile and standard error (SE).

Compound	IC_50_ Values (µM)			
	PI3Kα	PI3Kβ	PI3Kγ	PI3Kδ
**9a**	0.13 (SE ± 0.03)	1.99 (SE ± 0.79)	>10	1.22 (SE ± 0.47)
**9b**	0.63 (SE ± 0.13)	>10	>10	>10
**9c**	1.79 (SE ± 0.47)	>10	2.18 (SE ± 0.51)	1.37 (SE ± 0.37)
**9d**	0.63 (SE ± 0.15)	2.34 (SE ± 0.54)	1.64 (SE ± 0.29)	1.09 (SE ± 0.19)
**9e**	0.83 (SE ± 0.15)	6.48 (SE ± 1.85)	2.91 (SE ± 0.56)	0.90 (SE ± 0.11)
**9f**	>10	>10	4.43 (SE ± 0.69)	1.33 (SE ± 0.19)
**9g**	1.23 (SE ± 0.14)	>10	1.56 (SE ± 0.27)	0.85 (SE ± 0.14)
**9h**	0.98 (SE ± 0.12)	>10	2.43 (SE ± 0.41)	0.71 (SE ± 0.13)

Subsequently, compound **9b** was analyzed on cell-based assay in order to evaluate its ability to inhibit the PI3Kα signaling pathway on cells. It is well established that insulin signaling is mostly regulated by PI3Kα [29]. The activated insulin receptor triggers PI3Kα activity mainly by binding and phosphorylating adaptor proteins of the IRS (insulin receptor substrate) family. Once activated, PI3Kα produces the PtdIns(3,4,5)P_3_ lipid product, which in turn mediates the activation of downstream effectors such as Akt. Thus, phosphorylation of Akt results as a key event of the PI3Kα activation. In order to define whether compound **9b** was able to inhibit PI3Kα activity and, consequently, the insulin signaling, NIH3T3 cells were stimulated with insulin in the presence or absence of different concentrations of compound **9b**. A dose response curve and an IC_50_ value were determined. Compound **9b** showed an IC_50_ value of 2.8 µM (SE ± 0.6), thus demonstrating its ability to inhibit PI3Kα in a cellular context as well.

Molecular modeling studies were performed in order to investigate and rationalize this interesting selectivity. A simple search in the Protein Data Bank reveals over 50 releases spanning over all four PI3K isoforms, with only isoforms α and γ available from human sources. Docking compounds to rationalize little differences in activity still remains a challenge [30]. However, in our case, compound **9b** shows a relevant difference in activity against PI3Kα and PI3Kγ.

**Figure 3:**
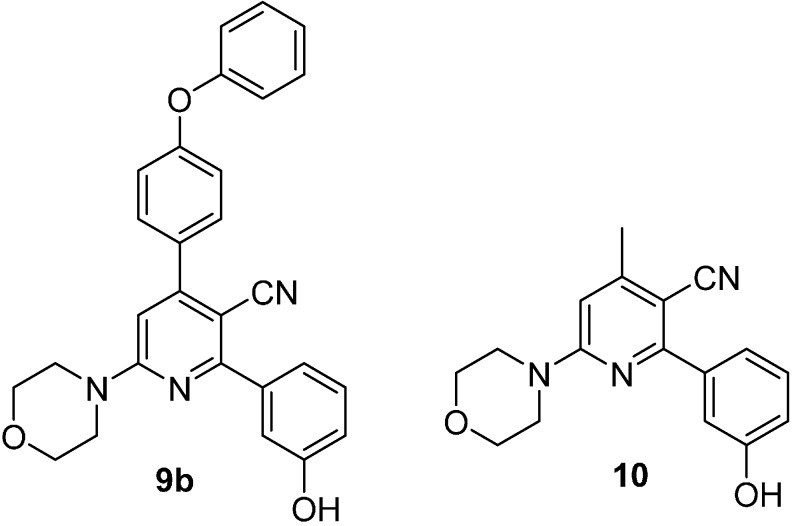
Compounds **9b** and **10** were docked on α and γ human PI3Ks.

In order to verify the importance of the aryl group at the C_4_ position to impart isoform selectivity, analogue **10**, where the aryl group has been replaced with a methyl group, was also considered and docked (Figure 3) (for the synthesis of **10** see supporting information).

Compound **10** is indeed a pan-inhibitor against the different isoforms (PI3Kα = 1.18 μM ± 0.17, PI3Kβ = 1.55 μM ± 0.30, PI3Kγ = 1.05 μM ± 0.10, and PI3Kδ = 0.42 μM ± 0.05).

Both compounds were prepared using Omega2 [31,32,33], and the docking software FRED [34,35] was used to predict their binding poses. The docking results for compound **10** (Supporting Information Table S1, Figure S1) showed a shared binding mode over the considered isoforms α and γ. The oxygen of morpholine is able to form a hydrogen bond interaction with the -NH of PI3Kγ Val882 (PI3Kα Val851) in the hinge region, while the phenolic group acts as hydrogen bond donor interacting with the –OH of Tyr867 in PI3Kγ (Tyr836 in PI3Kα). The methyl and cyano groups protrude toward the solvent-exposed region.

**Figure 4:**
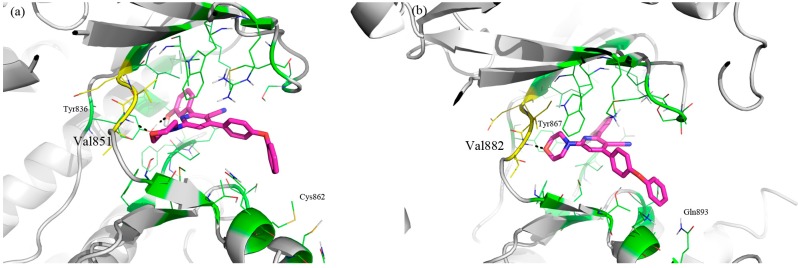
Predicted binding pose of compound **9b** (purple sticks) in the PI3Kα (**a**) and PI3Kγ (**b**) binding sites (green sticks and white cartoon); aminoacids of hinge region are shown as yellow sticks.

**Figure 5:**
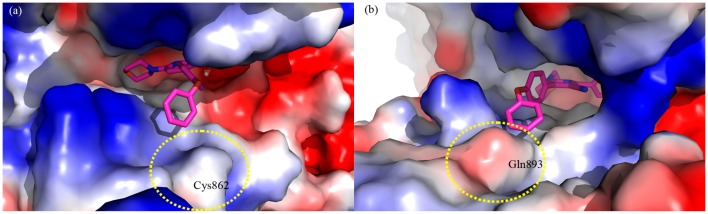
Details of surface electrostatic charges in PI3Kα (**a**) and PI3Kγ (**b**). The colors denote the electrostatic charge at the surface of the protein from negative (blue) to positive (red).

Poses of compound **9b** are consistent with docking results of **10** (Figure 4a,b). In this case, the aryl group protrudes toward the solvent-exposed region. The second aryl group of **9b** fits in a hydrophobic zone near Cys862 in PI3Kα, while in PI3Kγ a more polar environment is present. This is a consequence of the presence of Gln893 (Figure 5a,b). In PI3Kα the terminal oxydibenzene ring is positioned above an electrostatically positive area (Figure 5a), and this would be an attracting interaction with the electronegative π-cloud of the terminal ring of **9b**. In PI3Kγ the area below the terminal oxydibenzene ring is electrostatically negative and that would make for an unfavorable interaction (Figure 5b).

## 3. Experimental Section

### 3.1. Molecular Modeling

All molecular modeling studies were performed on a Tesla workstation equipped with two Intel Xenon X5650 2.67 GHz processors and Ubuntu 12.04 (www.ubuntu.com). All structural images were prepared using PyMOL [36] with the APBS plugin [37]. Different crystal structures of human PI3K isoforms with inhibitors have been reported; we select in our study the X-ray structures showing high resolution (<3 Å) and high chemical similarity of cocrystalized ligand to our compounds. The selected Protein Data Bank (PDB) id were: human PI3Kα (PDB id: 3ZIM, resolution 2.85 Å) [38], and human PI3Kγ (PDB id: 3DBS, res. 2.80 Å) [39]. Water molecules and ligands were removed; the binding site was detected using the original ligand coordinates (Supporting Information Figure S2). Hydrogens were added to the protein complexes, protonation and tautomer states were set, and finally the hydrogen bond network was optimized using the MolProbity server. [40]. Ligand structures were built from a SMILES string and were minimized using Omega2 [31,32,33]. A conformer library was built with the program Omega2, which generated an average of 81 conformations per molecule. Default parameters were used. The docking simulations were performed using FRED [34,35]. Two constraints were applied due to the presence of hydrogen bond between the original ligands and active pocket residues (referring PI3Kγ numbering: Val882, backbone amide hydrogen, and Tyr867, phenol oxygen). Default settings were used.

### 3.2. Chemistry

Synthetic procedures and spectral data are reported in the Supporting Information.

### 3.3. In Vitro Enzyme Inhibition

To evaluate the ability of each compound of formula (**9a**–**h**) to inhibit the lipid kinase activity of each PI3K isoform, 30 ng of PI3Kα, β, γ, and δ recombinant protein (purchased from JenaBioscience—Jena, Germany; PI3Kα #PR-335, PI3Kβ #PR-344, PI3Kγ #PR-343, PI3Kδ #PR-345) were incubated with at least five different concentrations (from 0.016 to 10 µM) of the compounds (**9a**–**h**). The phosphorylation reaction was then started by adding 10 μM of ATP, and 10 µg of lipid micelles containing the appropriate substrate phosphatidylinositol and the phosphatidylserine [41,42].

After 30 min of incubation (1 h for PI3Kβ recombinant protein) at room temperature, the reaction was stopped, and the amount of ADP metabolite was measured with a luminescent kinase assay, ADP-GloTM Kinase Assay (purchased from Promega—USA #V9101). The luminescent signal positively correlated with the formed ADP and, therefore, with the kinase activity. The percentage of kinase activity was calculated over the control samples, containing only dimethyl sulfoxide (DMSO). To derive the IC_50_ values for each assayed compound (**9a**–**h**), all data were plotted on a dose response curve (Graph Pad Software) and the IC_50_ was calculated by using the nonlinear regression fit (equation (log agonist) *vs.* response—Sigmoidal dose response). The IC_50_ was representative of two different experiments performed in triplicate.

### 3.4. Cell-Based Assay

NIH3T3 cells were cultured in Eagle's minimal essential medium (DMEM) (GIBCO-Thermo Fisher Scientific Inc., Waltham, MA, USA) supplemented with 10% Fetal Bovine Serum (FBS) (GIBCO-Thermo Fisher Scientific Inc.), 100 units/mL penicillin (GIBCO-Thermo Fisher Scientific Inc.), and 100 μg/mL streptomycin (GIBCO- Thermo Fisher Scientific Inc.). The day before the assay, NIH3T3 cells were seeded in a 96-well plate at a concentration of 5 × 104 cells/mL and starved O.N. in presence of DMEM 0.5% FBS. Subsequently, cells were pre-treated for 1.5 h with the inhibitor at different concentrations (From 0.03 µM to 20 µM). Then cells were stimulated for 5 min with DMEM containing 1 μM of insulin and proteins were extracted with Laemmli buffer. The P-Akt production is detected by Western blot analysis and quantified with ChemiDoc™ XRS system. From the absolute values, a percentage of residual P-Akt is calculated for each inhibitor concentration by using the control vehicle as 100%. To derive the IC_50_, all data are plotted on a dose response curve (Graph Pad software) and the IC_50_ is calculated by using the non-linear regression fit (equation (log agonist) *vs.* response—Sigmoidal dose response) on three different experiments.

## 4. Conclusions

In this manuscript, we described the use of 4-aryl-3-cyano-2-(3-hydroxyphenyl)-6-morpholino-pyridines as valuable starting points for the synthesis of PI3K inhibitors. We demonstrated that the modifications on the C_4_-position of this pyridine scaffold could impart a different profile of selectivity on the PI3K isoforms according to the pattern of substitution.

Compound **9b** was the most interesting of the series, with an activity profile selective for the PI3Kα isoform.

Molecular modeling studies tried to rationalize this profile of selectivity, pointing out that the presence of a polar Gln893 in the PI3Kγ isoform may disrupt the binding of **9b** through unfavorable electrostatic interactions.

Given these interesting results, further medicinal chemistry efforts are in progress to synthesize more structurally diverse analogues using the Guareschi reaction with the aim to increase the potency and identify different profiles of selectivity.

## Supplementary Materials

Supplementary materials can be accessed at: http://www.mdpi.com/1420-3049/20/09/17275/s1.
