# Supplementary material for: The Guareschi Pyridine Scaffold as a Valuable Platform for the Identification of Selective PI3K Inhibitors
Source: Molecules. 2015 Sep 18;20(9):17275–87. doi: 10.3390/molecules200917275 (PMC6332036; doi:10.3390/molecules200917275)
Supplement: Supplementary file 1 [file molecules-20-17275-s001.pdf]

# Supplementary Materials

## Experimental Procedures and Spectral Data

### Chemistry

Commercially available reagents and solvents were used without further purification and were purchased from Aldrich (Munich, Germany) or Alfa Aesar (Karlsruhe Germany). Toluene was distilled immediately before use from Na/benzophenone under a slight positive atmosphere of $N_2$. *N,N'*-dimethylformamide was distilled under vacuum from KOH and stored on activated molecular sieves (4 Å). When needed, the reactions were performed in flame- or oven-dried glassware under a positive pressure of dry $N_2$. Melting points were determined in an open glass capillary with a Stuart scientific SMP3 apparatus and are uncorrected. All compounds were checked by IR (FT-IR THERMO-NICOLET AVATAR, Waltham, MA, USA), $^{1}$H and $^{13}$C APT (JEOL ECP 300 MHz, Tokyo, Japan), and mass spectrometry (Thermo Finningan LCQ-deca XP-plus, Waltham, MA, USA) equipped with an ESI source and an ion trap detector. Chemical shifts are reported in part per million (ppm). Flash column chromatography was performed on silica gel (Merck Kieselgel 60, 230–400 mesh ASTM, Kenilworth, NJ USA). Thin layer chromatography (TLC) was carried out on 5 × 20 cm plates with a layer thickness of 0.25 mm (Merck Silica gel 60 $F_{254}$, Kenilworth, NJ, USA). When necessary, they were developed with $KMnO_4$ reagent. Purity of tested compounds was established by elemental analysis. Elemental analysis (C, H, N) of the target compounds are within ±0.4% of the calculated values.

### General Procedure A for the Preparation of Aryl-β-ketoesters (1a–h)

To a dried three-necked flask equipped with a dropping funnel, a condenser, and a magnetic stirrer, dimethyl carbonate **5** (2 eq), NaH 60% (2.8 eq) and dry toluene (2 M) were added. The mixture was heated to reflux under nitrogen. A solution of the corresponding aryl-methyl-ketone (**4a**–**h**) (1 eq) in dry toluene (2 M) was added dropwise over 1 h. The resulting reaction mixture was refluxed for 3 h, and then was cooled to 0 °C and glacial acetic acid was added dropwise until pH 4. The solid obtained was filtered and subsequently dissolved in hot water. The aqueous phase was extracted with EtOAc (×3). The combined organic layers were washed with brine, dried over sodium sulphate, and concentrated *in vacuo* to give the desired Aryl β-Keto ester, which was pure enough to be used for the next step without purification.

*Methyl 3-oxo-3-phenylpropanoate* (**1a**). The title compound was prepared from commercially available acetophenone (**4a**). Yellowish oil, yield 89%, IR (KBr) 3048, 1746, 1670 cm$^{-1}$, $^{1}$H-NMR (300 MHz, $CDCl_3$) (signals are referred to β-keto ester) δ 7.91 (d, J = 7.3 Hz, 2-H), 7.55 (t, *J* = 7.3 Hz, 1-H), 7.43 (t, *J* = 7.3 Hz, 2-H), 3.97 (s, 2-H), 3.70 (s, 3-H) ppm; $^{13}$C-NMR (75 MHz, $CDCl_3$) δ 192.4, 167.9, 135.8, 133.8, 128.8, 128.5, 52.4, 45.6 ppm. The spectral data were consistent with those reported previously [1].

*Methyl 3-oxo-3-(4-phenoxyphenyl)propanoate* (**1b**). The title compound was prepared from commercially available 1-(4-phenoxyphenyl)ethan-1-one (**4b**). Yellowish oil, yield 99%, IR (KBr) 3064, 2952, 1744, 1682, 1586, 1489, 1245, 1167, 871, 695 cm$^{-1}$. $^{1}$H-NMR (300 MHz, $CDCl_3$)

(signals are referred to β-keto ester) δ 7.91 (d, *J* = 8.9 Hz, 2-H), 7.38 (t, *J* = 7.7 Hz, 2-H), 7.20 (t, *J* = 7.3 Hz, 1-H), 7.06 (d, *J* = 7.3 Hz, 2-H), 6.98 (d, *J* = 8.9 Hz, 2-H), 3.95 (s, 2-H), 3.73 (s, 3-H) ppm; $^{13}$C-NMR (75 MHz, $CDCl_3$) δ 190.8, 168.0, 162.6, 155.1, 130.9, 130.5, 130.1, 124.9, 120.3, 117.3, 52.4, 45.5 ppm. The spectral data were consistent with those reported previously [2].

*Methyl 3-(4-methoxyphenyl)-3-oxopropanoate* (**1c**). The title compound was prepared from commercially available 1-(4-methoxyphenyl)ethan-1-one (**4c**). Yellowish oil, yield 98%, IR (KBr) 3080, 2927, 1741, 1670, 1603, 1324, 1148, 1025, 844 cm$^{-1}$. $^{1}$H-NMR (300 MHz, $CDCl_3$) (signals are referred to β-keto ester) δ 7.84 (d, *J* = 8.9 Hz, 2-H), 6.87 (d, *J* = 8.9 Hz, 2-H), 3.89 (s, 2-H), 3.78 (s, 3-H), 3.66 (s, 3-H) ppm; $^{13}$C-NMR (75 MHz, $CDCl_3$) δ 190.8, 168.1, 164.0, 130.8, 128.9, 113.9, 55.4, 52.2, 45.3 ppm. The spectral data were consistent with those reported previously [1].

*Methyl 3-(3-methoxyphenyl)-3-oxopropanoate* (**1d**). The title compound was prepared from commercially available 1-(3-methoxyphenyl)ethan-1-one (**4d**). Yellowish oil, yield 97%, IR (KBr) 2924, 2852, 1744, 1686, 1583, 1435, 1252, 1033, 788 cm$^{-1}$. $^{1}$H-NMR (300 MHz, $CDCl_3$) (signals are referred to β-keto ester) δ 7.48 (d, *J* = 7.3 Hz, 1-H), 7.47 (s, 1-H), 7.35 (t, *J* = 8.0 Hz, 1-H), 7.13–7.09 (m, 1-H), 3.97 (s, 2-H), 3.82 (s, 3-H), 3.73 (s, 3-H) ppm; $^{13}$C-NMR (75 MHz, $CDCl_3$) δ 192.1, 167.7, 160.1, 137.5, 129.8, 121.2, 120.4, 112.7, 55.5, 52.5, 45.8 ppm. The spectral data were consistent with those reported previously [1].

*Methyl 3-(3,4-dimethoxyphenyl)-3-oxopropanoate* (**1e**). The title compound was prepared from commercially available 1-(3,4-dimethoxyphenyl)ethan-1-one (**4e**). Orange oil, yield 98%, IR (KBr) 3629, 2954, 1743, 1674, 1516, 1274, 1152, 1022, 767 cm$^{-1}$. $^{1}$H-NMR (300 MHz, $CDCl_3$) (signals are referred to β-keto ester) δ 7.47–7.43 (m, 2-H), 6.81 (d, *J* = 8.3 Hz, 1-H), 3.88 (s, 2-H), 3.84 (s, 3-H), 3.83 (s, 3-H), 3.65 (s, 3-H) ppm; $^{13}$C-NMR (75 MHz, $CDCl_3$) δ 190.7, 168.0, 153.7, 149.1, 129.0, 123.4, 110.2, 110.0, 55.9, 55.8, 52.2, 45.2 ppm. The spectral data were consistent with those reported previously [3].

*Methyl 3-(3,4-dimethylphenyl)-3-oxopropanoate* (**1f**). The title compound was prepared from commercially available 1-(3,4-dimethylphenyl)ethan-1-one (**4f**). Orange oil, yield 97%, IR (KBr) 3630, 2952, 1744, 1682, 1607, 1445, 1326, 1210, 828 cm$^{-1}$. $^{1}$H-NMR (300 MHz, $CDCl_3$) (signals are referred to β-keto ester) δ 7.67 (s, 1-H), 7.62 (dd, *J* = 8.0/2.1 Hz, 1-H), 7.17 (d, *J* = 8.0 Hz, 1-H), 3.93 (s, 2-H), 3.70 (s, 3-H), 2.26 (s, 6-H) ppm; $^{13}$C-NMR (75 MHz, $CDCl_3$) δ 192.1, 168.1, 143.4, 137.1, 133.9, 129.9, 129.5, 126.3, 52.3, 45.5, 20.0 (2 C) ppm. The spectral data were consistent with those reported previously [4].

*Methyl 3-oxo-3-(pyridin-4-yl)propanoate* (**1g**). The title compound was prepared from commercially available 1-(pyridin-4-yl)ethan-1-one (**4g**). Orange oil, yield 96%, IR (KBr) 2923, 2853, 1659, 1459, 1377, 1264, 1207 cm$^{-1}$. $^{1}$H-NMR (300 MHz, $CDCl_3$) (signals are referred to enol form) δ 12.34 (br s, 1-H), 8.64 (dd, *J* = 4.6/1.5 Hz, 2-H), 7.55 (dd, *J* = 4.6/1.7 Hz, 2-H), 5.71 (s, 1-H), 3.74 (s, 3-H) ppm; $^{13}$C-NMR (75 MHz, $CDCl_3$) δ 172.9, 168.2, 150.1, 140.9, 119.8, 89.6, 51.6 ppm. The spectral data were consistent with those reported previously [5].

*Methyl 3-oxo-3-(pyridin-3-yl)propanoate* (**1h**). The title compound was prepared from commercially available 1-(pyridin-3-yl)ethan-1-one (**4h**). Orange oil, yield 98%, IR (KBr) 3606, 2923, 1743, 1692, 1587, 1274, 1214, 803, 703 $cm^{-1}$. $^{1}$H-NMR (300 MHz, $CDCl_3$) (signals are referred to β-keto ester) δ 9.12 (s, 1-H), 8.79–8.77 (m, 1-H), 8.24–8.20 (m, 1-H), 7.46–7.41 (m, 1-H), 4.00 (s, 2-H), 3.72 (s, 3-H) ppm; $^{13}$C-NMR (75 MHz, $CDCl_3$) δ 191.3, 167.3, 153.8, 149.7, 136.1, 131.5, 124.0, 52.7, 45.7 ppm. The spectral data were consistent with those reported previously [6].

**General Procedure B for the Preparation of 4-Aryl-3-cyano-2,6-dihydroxypyridines (3a–h)**

Cyanoacetamide **2** (1 eq) and the corresponding Aryl β-keto ester (**1a**–**h**) (1 eq) were dissolved in methanol (4 M). To the vigorously stirred and refluxed mixture KOH (1.2 eq) in methanol (24 M) was added dropwise over 1 h. During the addition, a white precipitate formed and enough methanol was added to prevent caking. The resulting reaction mixture was refluxed overnight, then was cooled to 0 °C to induce precipitation of the product. The 3-cyano-2,6-dihydroxy-4-arylpyridine monopotassium salt was collected by filtration, washed with methanol, and subsequently dissolved in hot water. The solution was cooled (0 °C) and acidified with concentrated hydrochloric acid until pH 1. The desired solid was separated by filtration, washed with methanol, water, and dried *in vacuo.* The product was pure enough to be used for the next step without purification.

*2,6-Dihydroxy-4-phenylnicotinonitrile* (**3a**). The title compound was prepared from **1a**. Off-white solid, yield 41%, m.p. 279–280 °C dec. IR (KBr) 2923, 2853, 2215, 1592, 1460, 1373, 1279, 700 $cm^{-1}$. $^{1}$H-NMR (300 MHz, $(CD_3)_2SO$) δ 7.51–7.49 (m, 5-H), 5.62 (br s, 1-H) ppm; $^{13}$C-NMR (75 MHz, $(CD_3)_2SO$) δ 162.7, 161.7, 160.8, 137.0, 130.0, 128.8, 127.8, 117.7, 92.7, 87.2 ppm; MS (ESI) *m*/*z* 211 $(M - H)^-$. The spectral data were consistent with those reported previously [7].

*2,6-Dihydroxy-4-(4-phenoxyphenyl)nicotinonitrile* (**3b**). The title compound was prepared from **1b**. Off-white solid, yield 15%, m.p. 302–303 °C dec. IR (KBr) 3076, 2928, 2198, 1637, 1608, 1490, 1369, 1249, 754 $cm^{-1}$. $^{1}$H-NMR (300 MHz, $(CD_3)_2SO$) δ 9.78 (s, 1-H), 7.46–7.40 (m, 4-H), 7.18 (t, $J$ = 7.3 Hz, 1-H), 7.08 (d, $J$ = 7.6 Hz, 2-H), 7.00 (d, $J$ = 8.6 Hz, 2-H), 5.00 (s, 1-H) ppm; $^{13}$C-NMR (75 MHz, $(CD_3)_2SO$) δ 165.6, 164.4, 157.0, 156.1, 155.4, 134.4, 130.2, 129.3, 123.9, 121.8, 119.2, 118.2, 117.7, 98.8 ppm.

*2,6-Dihydroxy-4-(4-methoxyphenyl)nicotinonitrile* (**3c**). The title compound was prepared from **1c**. Off-white solid, yield 14%, m.p. 289–291 °C dec. IR (KBr) 3083, 2923, 2218, 1603, 1514, 1300, 1183, 1023, 823 $cm^{-1}$. $^{1}$H-NMR (300 MHz, $(CD_3)_2SO$) δ 7.51 (d, $J$ = 8.6 Hz, 2-H), 7.07 (d, $J$ = 8.8 Hz, 2-H), 5.67 (s, 1-H), 3.82 (s, 3-H) ppm; $^{13}$C-NMR (75 MHz, $(CD_3)_2SO$) δ 162.7, 161.3, 160.6, 160.2, 129.3, 128.9, 117.8, 114.1, 95.4, 86.8, 55.3 ppm; MS (ESI) *m*/*z* 241 $(M - H)^-$. The spectral data were consistent with those reported previously [8].

*2,6-Dihydroxy-4-(3-methoxyphenyl)nicotinonitrile* (**3d**). The title compound was prepared from **1d**. Off-white solid, yield 28%, m.p. 299–300 °C dec. IR (KBr) 3348, 3204, 2926, 2195, 1637, 1603, 1369, 1047, 753 $cm^{-1}$. $^{1}$H-NMR (300 MHz, $(CD_3)_2SO$) δ 7.31 (t, $J$ = 7.9 Hz, 1-H), 7.00–6.92 (m, 3-H), 5.06

(s, 1-H), 3.78 (s, 3-H) ppm; $^{13}$C-NMR (75 MHz, $(CD_3)_2SO$) δ 165.4, 164.2, 158.9, 156.4, 140.7, 129.3, 121.4, 119.8, 114.0, 113.0, 98.4, 87.2, 55.1 ppm.

*4-(3,4-Dimethoxyphenyl)-2,6-dihydroxynicotinonitrile* (**3e**). The title compound was prepared from **1e**. Off-white solid, yield 9%, m.p. 279–280 °C dec. IR (KBr) 3347, 3204, 2938, 2194, 1712, 1638, 1604, 753 $cm^{-1}$. $^{1}$H-NMR (300 MHz, $(CD_3)_2SO$) δ 7.15–7.06 (m, 3-H), 5.69 (s, 1-H), 3.81 (br s, 6-H) ppm; $^{13}$C-NMR (75 MHz, $(CD_3)_2SO$) δ 165.8, 164.5, 156.1, 149.0, 148.1, 131.9, 122.0, 120.0, 116.4, 111.6, 111.4, 98.6, 55.6, 55.5 ppm.

*4-(3,4-Dimethylphenyl)-2,6-dihydroxynicotinonitrile* (**3f**). The title compound was prepared from **1f**. White solid, yield 27%, m.p. 310–312 °C dec. IR (KBr) 3074, 2734, 2223, 1625, 1589, 1551, 1308, 861 $cm^{-1}$. $^{1}$H-NMR (300 MHz, $(CD_3)_2SO$) δ 7.30 (s, 1-H), 7.26 (s, 2-H), 5.66 (s, 1-H), 2.28 (s, 6-H) ppm; $^{13}$C-NMR (75 MHz, $(CD_3)_2SO$) δ 162.7, 161.4, 160.8, 138.4, 136.7, 134.5, 129.7, 128.6, 125.1, 117.6, 92.3, 87.0, 19.4, 19.2 ppm.

*2,6-Dihydroxy-[4,4'-bipyridine]-3-carbonitrile* (**3g**). The title compound was prepared from **1g**. Yellow solid, yield 26%, m.p. 331–333 °C dec. IR (KBr) 3061, 2199, 1613, 1519, 1397, 814 $cm^{-1}$. $^{1}$H-NMR (300 MHz, $(CD_3)_2SO$) δ 8.77 (br d, 2-H), 7.69 (br d, 2-H), 5.31 (s, 1-H) ppm; $^{13}$C-NMR (75 MHz, $(CD_3)_2SO$) δ 157.2, 152.6, 149.8 (2c), 148.5, 144.4, 123.8 (2c), 114.6, 84.0, 83.9 ppm. MS (ESI) *m/z* 212 $(M - H)^{-}$.

*2',6'-Dihydroxy-[3,4'-bipyridine]-3'-carbonitrile* (**3h**). The title compound was prepared from **1h**. Yellowish solid, yield 10%, m.p. 289–291 °C dec. IR (KBr) 3392, 3113, 2923, 2194, 1638, 1583, 1382, 814, 679 $cm^{-1}$. $^{1}$H-NMR (300 MHz, $(CD_3)_2SO$) δ 8.78 (s, 1-H), 8.73 (d, $J$ = 4.3 Hz, 1-H), 8.09 (dt, $J$ = 8.0/1.8 Hz, 1-H), 7.65 (dd, $J$ = 7.6/5.2 Hz, 1-H), 5.5 (s, 1-H) ppm; $^{13}$C-NMR (75 MHz, $(CD_3)_2SO$) δ 163.5, 163.0, 155.2, 148.7, 146.4, 137.9, 134.5, 124.6, 118.7, 95.4, 83.2 ppm.

**General Procedure C for the Preparation of 4-Aryl-3-cyano-2,6-dichloropyridines (6a–h)**

A mixture of the corresponding 4-aryl-3-cyano-2,6-dihydroxypyridine (**3a**–**h**) (1 eq) and $POCl_3$ (10 eq) was heated and stirred in a Carius tube at 140 °C overnight. After cooling to room temperature, the reaction mixture was transferred cautiously and with stirring onto water and ice. The resulting aqueous phase was extracted with dichloromethane (×3). The combined organic layers were washed with brine, dried over sodium sulphate and concentrated *in vacuo*. The crude material was purified by flash column chromatography using PE/EtOAc 9:1 and PE/EtOAc 8:2 as eluants to give the desired product.

*2,6-Dichloro-4-phenylnicotinonitrile* (**6a**). The title compound was prepared from **3a**. Off-white solid, yield 67%, m.p. 166–168 °C. IR (KBr) 3068, 2923, 2853, 2231, 1567, 1520, 1339, 1130, 852, 778 $cm^{-1}$. $^{1}$H-NMR (300 MHz, $CDCl_3$) δ 7.57–7.56 (m, 5-H), 7.44 (s, 1-H) ppm; $^{13}$C-NMR (75 MHz, $CDCl_3$) δ 163.8, 157.9, 153.9, 134.0, 131.3, 129.5, 128.5, 123.4, 114.2, 108.2 ppm. The spectral data were consistent with those reported previously [7].

*2,6-Dichloro-4-(4-phenoxyphenyl)nicotinonitrile* (**6b**). The title compound was prepared from **3b**. Yellowish solid, yield 49%, m.p. 136–137 °C. IR (KBr) 3069, 2924, 2230, 1508, 1489, 1259, 1066,

834, 693 $cm^{-1}$. $^1$H-NMR (300 MHz, $CDCl_3$) δ 7.57 (d, *J* = 8.6 Hz, 2-H), 7.41 (t, *J* = 7.9 Hz, 3-H), 7.21 (t, *J* = 7.0 Hz, 1-H), 7.13–7.08 (m, 4-H) ppm; $^{13}$C-NMR (75 MHz, $CDCl_3$) δ 160.7 (2-C), 157.2, 155.5, 153.9, 130.3, 130.2, 127.9, 124.8, 123.0, 120.3, 118.4, 114.4, 107.8 ppm.

*2,6-Dichloro-4-(4-methoxyphenyl)nicotinonitrile* (**6c**). The title compound was prepared from **3c**. White solid, yield 7%, m.p. 171–172 °C. IR (KBr) 3009, 2846, 2225, 1509, 1520, 1264, 1184, 836 $cm^{-1}$; $^1$H-NMR (300 MHz, $CDCl_3$) δ 7.57 (d, *J* = 8.9 Hz, 2-H), 7.39 (s, 1-H), 7.04 (d, *J* = 8.9 Hz, 2-H), 3.88 (s, 3-H) ppm; $^{13}$C-NMR (75 MHz, $CDCl_3$) δ 162.1, 157.4, 154.0, 153.7, 130.2, 126.0, 122.9, 114.9, 114.6, 107.5, 55.6 ppm. M*S* (ESI) *m*/*z* 302 $(M + Na)^+$.

*2,6-Dichloro-4-(3-methoxyphenyl)nicotinonitrile* (**6d**). The title compound was prepared from **3d**. Pale yellowish solid, yield 25%, m.p. 168–170 °C. IR (KBr) 2923, 2853, 2230, 1570, 1525, 1338, 1288, 1045, 793 $cm^{-1}$. $^1$H-NMR (300 MHz, $CDCl_3$) δ 7.45–7.42 (m, 2-H), 7.13 (dt, *J* = 8.0/1.2 Hz, 1-H), 7.09–7.07 (m, 2-H), 3.86 (s, 3-H) ppm; $^{13}$C-NMR (75 MHz, $CDCl_3$) δ 160.2, 157.8, 154.0, 153.9, 135.2, 130.7, 123.5, 120.7, 117.0, 114.2, 113.9, 108.2, 55.0 ppm.

*2,6-Dichloro-4-(3,4-dimethoxyphenyl)nicotinonitrile* (**6e**). The title compound was prepared from **3e**. Yellow solid, yield 41%, m.p. 148–150 °C. IR (KBr) 2924, 2854, 2225, 1523, 1509, 1266, 1143, 854 $cm^{-1}$. $^1$H-NMR (300 MHz, $CDCl_3$) δ 7.40 (s, 1-H), 7.19 (dd, *J* = 8.6/2.2 Hz, 1-H), 7.12 (d, *J* = 2.1 Hz, 1-H), 6.98 (d, *J* = 8.6 Hz, 1-H), 3.93 (s, 6-H) ppm; $^{13}$C-NMR (75 MHz, $CDCl_3$) δ 157.4, 154.0, 153.7, 151.7, 149.5, 126.1, 122.8, 121.9, 114.6, 111.6, 111.3, 107.6, 56.3, 56.2 ppm.

*2,6-Dichloro-4-(3,4-dimethylphenyl)nicotinonitrile* (**6f**). The title compound was prepared from **3f**. White solid, yield 64%, m.p. 158–160 °C. IR (KBr) 2927, 2850, 2230, 1520, 1335, 1264, 855 $cm^{-1}$. $^1$H-NMR (300 MHz, $CDCl_3$) δ 7.40 (s, 1-H), 7.35–7.30 (m, 3-H), 2.34 (br s, 6-H) ppm; $^{13}$C-NMR (75 MHz, $CDCl_3$) δ 160.0, 153.7, 153.6, 140.5, 137.9, 131.4, 130.6, 129.4, 125.9, 123.1, 114.4, 107.9, 19.9, 19.8 ppm.

*2,6-Dichloro-[4,4'-bipyridine]-3-carbonitrile* (**6g**). The title compound was prepared from **3g**. Off white solid, yield 82%, m.p. 169–170 °C dec. IR (KBr) 3075, 2233, 1567, 1519, 1346, 1324, 1136, 826 $cm^{-1}$. $^1$H-NMR (300 MHz, $CDCl_3$) δ 8.84 (d, *J* = 4.8 Hz, 2-H), 7.49 (d, *J* = 4.6 Hz, 2-H), 7.44 (s, 1-H) ppm; $^{13}$C-NMR (75 MHz, $CDCl_3$) δ 154.8, 154.6, 154.3, 150.9, 141.6, 123.2, 122.5, 113.4, 108.1 ppm.

*2',6'-Dichloro-[3,4'-bipyridine]-3'-carbonitrile* (**6h**). The title compound was prepared from **3h**. Off-white solid, yield 74%, m.p. 152–154 °C dec. IR (KBr) 3036, 2923, 2231, 1560, 1522, 1342, 814, 713 $cm^{-1}$. $^1$H-NMR (300 MHz, $CDCl_3$) δ 8.81 (br s, 2-H), 7.98 (dt, *J* = 8.0/2.1 Hz, 1-H), 7.52 (dd, *J* = 8.0/4.9 Hz, 1-H), 7.46 (s, 1-H) ppm; $^{13}$C-NMR (75 MHz, $CDCl_3$) δ 154.5, 154.4, 154.2, 152.2, 148.7, 135.9, 130.2, 123.9, 123.3, 113.7, 108.4 ppm.

**General Procedure D for the Preparation of 4-Aryl-2-chloro-3-cyano-6-(morpholino-4-yl)pyridines (7a–h)**

Under a nitrogen atmosphere, morpholine (2.2 eq) was added dropwise to a stirred solution of the corresponding 4-aryl-3-cyano-2,6-dichloropyridine (**6a**–**h**) (1 eq) in methanol (0.7 M) at 0 °C. The mixture was vigorously stirred at 25 °C overnight. After completion of the reaction, the resulting suspension was filtered. The solid was washed with cold methanol and cold water. Finally, the desired product was dried *in vacuo* and used for the next step without further purification.

*2-Chloro-6-morpholino-4-phenylnicotinonitrile* (**7a**). The title compound was prepared from **6a**. Off-white solid, yield 73%, m.p. 205–206 °C. IR (KBr) 2972, 2922, 2866, 2216, 1594, 1500, 1257, 1121, 972, 693 $cm^{-1}$. $^{1}$H-NMR (300 MHz, $CDCl_3$) δ 7.50–7.46 (m, 5-H), 6.46 (s, 1-H), 3.80 (t, $J$ = 4.9 Hz, 4-H), 3.68 (t, $J$ = 4.9 Hz, 4-H) ppm; $^{13}$C-NMR (75 MHz, $CDCl_3$) δ 158.5, 156.3, 153.9, 136.6, 130.1, 129.0, 128.3, 116.5, 104.3, 96.3, 66.5, 45.0 ppm.

*2-Chloro-6-morpholino-4-(4-phenoxyphenyl)nicotinonitrile* (**7b**). The title compound was prepared from **6b**. White solid, yield 76%, m.p. 155–156 °C. IR (KBr) 2962, 2867, 2212, 1590, 1489, 1249, 1201, 973, 838 $cm^{-1}$. $^{1}$H-NMR (300 MHz, $CDCl_3$) δ 7.49 (d, $J$ = 8.9 Hz, 2-H), 7.38 (t, $J$ = 7.6 Hz, 2-H), 7.17 (t, $J$ = 7.3 Hz, 1-H), 7.09–7.05 (m, 4-H), 6.44 (s, 1-H), 3.80 (t, $J$ = 5.2 Hz, 4-H), 3.70 (t, $J$ = 5.2 Hz, 4-H) ppm; $^{13}$C-NMR (75 MHz, $CDCl_3$) δ 159.4, 158.5, 156.0, 155.5, 153.9, 130.8, 130.1, 129.9, 124.3, 119.9, 118.3, 116.7, 104.0, 95.9, 66.5, 50.0 ppm.

*2-Chloro-4-(4-methoxyphenyl)-6-morpholinonicotinonitrile* (**7c**). The title compound was prepared from **6c**. White solid, yield 56%, m.p. 219–220 °C. IR (KBr) 2968, 2838, 2211, 1592, 1519, 1443, 1254, 1116, 973, 835 $cm^{-1}$. $^{1}$H-NMR (300 MHz, $CDCl_3$) δ 7.49 (d, $J$ = 8.9 Hz, 2-H), 6.99 (d, $J$ = 8.9 Hz, 2-H), 6.43 (s, 1-H), 3.85 (s, 3-H), 3.81–3.78 (m, 4-H), 3.69–3.66 (m, 4-H) ppm; $^{13}$C-NMR (75 MHz, $CDCl_3$) δ 161.3, 158.7, 156.0, 154.0, 129.8, 129.0, 116.7, 114.6, 103.9, 96.4, 66.5, 55.6, 45.2 ppm.

*2-Chloro-4-(3-methoxyphenyl)-6-morpholinonicotinonitrile* (**7d**). The title compound was prepared from **6d**. Yellowish solid, yield 62%, m.p. 147–148 °C. IR (KBr) 2923, 2853, 2218, 1588, 1458, 1254, 1122, 978 $cm^{-1}$. $^{1}$H-NMR (300 MHz, $CDCl_3$) δ 7.38 (br s, 1-H), 7.09–7.02 (m, 3-H), 6.47 (s, 1-H), 3.84–3.78 (m, 8-H), 3.68 (s, 3-H) ppm; $^{13}$C-NMR (75 MHz, $CDCl_3$) δ 159.8, 158.4, 156.0, 153.8, 137.8, 130.1, 120.5, 116.5, 115.6, 113.8, 104.2, 96.1, 66.4, 55.5, 44.9 ppm.

*2-Chloro-4-(3,4-dimethoxyphenyl)-6-morpholinonicotinonitrile* (**7e**). The title compound was prepared from **6e**. White solid, yield 50%, m.p. 196–199 °C. IR (KBr) 2850, 2210, 1600, 1522, 1491, 1258, 1168, 760 $cm^{-1}$. $^{1}$H-NMR (300 MHz, $CDCl_3$) δ 7.08 (dd, $J$ = 8.3/2.1 Hz, 1-H), 7.05 (s, 1-H), 6.92 (d, $J$ = 8.3 Hz, 1-H), 6.44 (s, 1-H), 3.91 (br s, 6-H), 3.77 (t, $J$ = 4.3 Hz, 4-H), 3.66 (t, $J$ = 4.0 Hz, 4-H) ppm.$^{13}$C-NMR (75 MHz, $CDCl_3$) δ 158.8, 156.2, 154.2, 150.9, 149.4, 129.3, 121.5, 117.2, 111.7, 111.6, 104.1, 96.3, 66.8, 56.5, 56.4, 45.3 ppm.

*2-Chloro-4-(3,4-dimethylphenyl)-6-morpholinonicotinonitrile* (**7f**). The title compound was prepared from **6f**. White solid, yield 80%, m.p. 188–190 °C dec. IR (KBr) 2977, 2896, 2217, 1595, 1488, 1246,

1122, 975, 817 $cm^{-1}$. $^{1}$H-NMR (300 MHz, $CDCl_3$) δ 7.27–7.24 (m, 3-H), 6.44 (s, 1-H), 3.79 (t, $J$ = 4.9 Hz, 4-H), 3.67 (t, $J$ = 4.9 Hz, 4-H), 2.32 (br s, 6-H) ppm; $^{13}$C-NMR (75 MHz, $CDCl_3$) δ 158.6, 156.5, 153.8, 139.0, 137.4, 134.2, 130.3, 129.3, 125.7, 116.7, 104.1, 96.4, 66.6, 45.1, 20.0, 19.8 ppm.

*2-Chloro-6-morpholino-[4,4'-bipyridine]-3-carbonitrile* (**7g**). The title compound was prepared from **6g**. Yellowish solid, yield 42%, m.p. 202–203 °C dec. IR (KBr) 3009, 2955, 2854, 2212, 1589, 1492, 1245, 1111, 821 $cm^{-1}$. $^{1}$H-NMR (300 MHz, $CDCl_3$) δ 8.70 (d, $J$ = 4.9 Hz, 2-H), 7.41 (d, $J$ = 4.9 Hz, 2-H), 6.44 (s, 1-H), 3.76 (br s, 4-H), 3.68 (br s, 4-H) ppm; $^{13}$C-NMR (75 MHz, $CDCl_3$) δ 158.3, 154.1, 153.0, 150.3, 144.2, 122.6, 115.7, 104.0, 95.1, 66.3, 45.0 ppm.

*2'-Chloro-6'-morpholino-[3,4'-bipyridine]-3'-carbonitrile* (**7h**). The title compound was prepared from **6h**. White solid, yield 60%, m.p. 209–211 °C dec. IR (KBr) 3078, 2922, 2217, 1600, 1494, 1122, 970, 861, 714 $cm^{-1}$. $^{1}$H-NMR (300 MHz, $CDCl_3$) δ 8.72 (br s, 2-H), 7.91 (dt, $J$ = 8.0/1.8 Hz, 1-H), 7.43 (dd, $J$ = 8.0/4.9 Hz, 1-H), 6.46 (s, 1-H), 3.79 (t, $J$ = 4.3 Hz, 4-H), 3.70 (t, $J$ = 4.3 Hz, 4-H) ppm; $^{13}$C-NMR (75 MHz, $CDCl_3$) δ 158.4, 154.0, 152.6, 151.0, 148.6, 135.8, 132.6, 123.6, 116.0, 104.2, 95.8, 66.4, 45.1 ppm.

**General Procedure E for the Preparation of 4-Aryl-3-cyano-2-(3-hydroxyphenyl)-6-(morpholino-4-yl)pyridines (9a–h)**

Under a nitrogen atmosphere, 3-hydroxyphenylboronic acid (**8)** (1.1 eq), $Na_2CO_3$ (3 eq) (dissolved in the minimum quantity of water) and $Pd(PPh_3)_4$ (0.1 eq) were added to a stirred solution of the corresponding intermediate **7a**–**h** (1 eq) in DMF (0.35 M). The reaction was heated at 100 °C overnight. After completion, the resulting mixture was diluted with water and extracted with EtOAc (×3). The combined organic layers were washed with brine, dried over sodium sulphate, and concentrated *in vacuo*. The crude material was purified by flash column chromatography.

*2-(3-Hydroxyphenyl)-6-morpholino-4-phenylnicotinonitrile* (**9a**). The title compound was prepared from **7a**. The crude material was purified by column chromatography using PE/EtOAc 95:5 and PE/EtOAc 7:3 as eluants, to give a white solid, yield 52%, m.p. 221–223 °C dec. IR (KBr) 3238, 2885, 2210, 1586, 1577, 1456, 1247, 1114, 696 $cm^{-1.}$ $^{1}$H-NMR (300 MHz, $(CD_3)_2SO$) δ 9.67 (s, 1-H), 7.66–7.63 (m, 2-H), 7.56–7.53 (m, 3-H), 7.28–7.23 (m, 3-H), 6.90 (s, 1-H), 3.72 (br s, 8-H) ppm; $^{13}$C-NMR (75 MHz, $(CD_3)_2SO$) δ 161.6, 158.4, 157.2, 154.4, 139.7, 137.5, 129.4, 129.3, 128.7, 128.6, 119.7, 118.9, 116.7, 115.8, 105.2, 92.9, 66.0, 44.5 ppm. MS (ESI) *m/z* 380 $(M + Na)^+$. Anal. Calcd for $C_{22}H_{19}N_3O_2$: C, 73.93; H, 5.36; N, 11.76; Found: C, 74.15; H, 5.68; N, 11.96.

*2-(3-Hydroxyphenyl)-6-morpholino-4-(4-phenoxyphenyl)nicotinonitrile* (**9b**). The title compound was prepared from **7b**. The crude material was purified by column chromatography using PE/EtOAc 8:2 and PE/EtOAc 7:3 as eluants, to give a white solid, yield 43%, m.p. 240–242 °C. IR (KBr) 3339, 2963, 2861, 2216, 1574, 1508, 1488, 1246, 980 $cm^{-1}$. $^{1}$H-NMR (300 MHz, $(CD_3)_2SO$) δ 9.6 (s, 1-H), 7.68 (d, $J$ = 8.6 Hz, 2-H), 7.46 (t, $J$ = 8.0 Hz, 2-H), 7.31–7.20 (m, 4-H), 7.14–7.11 (m, 4-H), 6.90 (br s, 2-H), 3.72 (br s, 8-H) ppm; $^{13}$C-NMR (75 MHz, $(CD_3)_2SO$) δ 158.4, 158.2, 157.3, 153.7, 151.3, 139.8, 132.2, 130.7, 130.4, 129.3, 128.4, 124.3, 119.7, 119.5, 119.0, 118.0, 116.7, 115.8, 105.0, 98.5, 66.0,

44.5 ppm.MS (ESI) *m/z* 450 (M + H)$^+$. Anal. Calcd for $C_{28}H_{23}N_3O_3$: C, 74.82; H, 5.16; N, 9.35; Found: C, 74.74; H, 4.94; N, 9.64.

*2-(3-Hydroxyphenyl)-4-(4-methoxyphenyl)-6-morpholinonicotinonitrile* (**9c**). The title compound was prepared from **7c**. The crude material was purified by column chromatography using PE/EtOAc 8:2 and PE/EtOAc 7:3 as eluants, to give a white solid which was crystallized with EtOAc, yield 17%, m.p. 216–217 °C. IR (KBr) 3434, 2961, 2867, 2201, 1581, 1517, 1253, 1113, 829 cm$^{-1}$. $^1$H-NMR (300 MHz, $CDCl_3$) δ 7.93 (d, *J* = 6.7 Hz, 1-H), 7.53–7.46 (m, 3-H), 7.37–7.25 (m, 2-H), 7.00 (d, *J* = 8.3 Hz, 2-H), 6.52 (s, 1-H), 3.86–3.73 (m, 11-H) ppm; $^{13}$C-NMR (75 MHz, $CDCl_3$) δ 162.1, 160.9, 158.9, 155.8, 155.5, 140.2, 130.0, 129.7, 121.8, 119.3, 119.2, 117.1, 116.3, 114.4, 104.5, 94.7, 66.7, 55.6, 45.1 ppm. MS (ESI) *m/z* 388 (M + H)$^+$. Anal. Calcd for $C_{23}H_{21}N_3O_3$: C, 71.30; H, 5.46; N, 10.85; Found: C, 71.67; H, 5.78; N, 10.73.

*2-(3-Hydroxyphenyl)-4-(3-methoxyphenyl)-6-morpholinonicotinonitrile* (**9d**). The title compound was prepared from **7d**. The crude material was purified by column chromatography using PE/EtOAc 8:2 and PE/EtOAc 6:4 as eluants, to give a white solid which was crystallized with EtOAc, yield 62%, m.p. 208–209 °C dec. IR (KBr) 3305, 2872, 2846, 2203, 1576, 1442, 1231, 1105, 878 cm$^{-1}$. $^1$H-NMR (300 MHz, $(CD_3)_2SO$) δ 9.69 (s, 1-H), 7.45 (t, *J* = 8.3 Hz, 1-H), 7.31 (m, 5-H), 7.09 (dd, *J* = 7.7/1.9 Hz, 1-H), 6.91–6.89 (m, 2-H), 3.83 (s, 3-H), 3.73 ( br s, 8-H) ppm; $^{13}$C-NMR (75 MHz, $(CD_3)_2SO$) δ 161.6, 159.3, 158.4, 157.3, 154.2, 139.8, 138.8, 129.8, 129.3, 121.0, 119.7, 118.9, 116.7, 115.8, 115.0, 114.4, 105.1, 92.9, 65.9, 55.4, 44.6 ppm. MS (ESI) *m/z* 388 (M + H)$^+$. Anal. Calcd for $C_{23}H_{21}N_3O_3$: C, 71.30; H, 5.46; N, 10.85; Found: C, 71.64; H, 5.63; N, 11.03.

*4-(3,4-Dimethoxyphenyl)-2-(3-hydroxyphenyl)-6-morpholinonicotinonitrile* (**9e**). The title compound was prepared from **7e**. The crude material was purified by column chromatography using PE/EtOAc 7:3 and PE/EtOAc 6:4 as eluants, to give a white solid which was crystallized with PE, yield 36%, m.p. 142–144 °C. IR (KBr) 3236, 2963, 2210, 1581, 1518, 1449, 1262, 1232, 1111, 886 cm$^{-1}$, $^1$H-NMR (300 MHz, $CDCl_3$) δ 7.46 (d, *J* = 8.3 Hz, 1-H), 7.37 (t, *J* = 2.2 Hz, 1-H), 7.30 (t, *J* = 8.0 Hz, 1-H), 7.15–7.10 (m, 2-H), 6.96–6.90 (m, 2-H), 6.54 (s, 1-H), 3.93 (s, 3-H), 3.91 (s, 3-H), 3.80 (t, *J* = 5.2 Hz, 4-H), 3.72 (t, *J* = 5.2 Hz, 4-H) ppm; $^{13}$C-NMR (75 MHz, $CDCl_3$) δ 162.6, 159.1, 156.5, 155.7, 150.6, 149.3, 140.2, 130.6, 130.0, 121.8, 121.7, 119.9, 117.5, 116.6, 112.3, 111.7, 104.7, 94.7, 67.0, 56.6, 56.5, 45.3 ppm. MS (ESI) *m/z* 418 (M + H)$^+$. Anal. Calcd for $C_{24}H_{23}N_3O_4$: C, 69.05; H, 5.55; N, 10.07; Found: C, 69.12; H, 5.56; N, 10.32.

*4-(3,4-Dimethylphenyl)-2-(3-hydroxyphenyl)-6-morpholinonicotinonitrile* (**9f**). The title compound was prepared from **7f**. The crude material was purified by column chromatography using PE/EtOAc 9:1 and PE/EtOAc 7:3 as eluants, to give a white solid, yield 52%, m.p. 117–120 °C dec. IR (KBr) 3345, 2857, 2211, 1580, 1443, 1248, 1115, 980, 891 cm$^{-1}$. $^1$H-NMR (300 MHz, $CDCl_3$) δ 7.49 (d, *J* = 7.6 Hz, 1-H), 7.37–7.30 (m, 4-H), 7.24 (d, *J* = 7.9 Hz, 1-H), 6.93 (dd, *J* = 7.9/1.8 Hz, 1-H), 6.54 (s, 1-H), 3.82 (t, *J* = 5.5 Hz, 4-H), 3.73 (t, *J* = 5.2 Hz, 4-H), 2.33 (s, 3-H), 2.32 (s, 3-H) ppm; $^{13}$C-NMR (75 MHz, $CDCl_3$) δ 158.7, 155.9, 155.8, 139.9, 138.4, 137.2, 135.3, 130.1, 129.7, 129.6, 126.0, 121.6, 119.3, 117.1, 116.1, 104.5, 92.9, 66.7, 44.9, 19.9, 19.8 ppm. MS (ESI) *m/z* 386 (M + H)$^+$. Anal. Calcd for $C_{24}H_{23}N_3O_2$: C, 74.78; H, 6.01; N, 10.90; Found: C, 74.85; H, 6.32; N, 10.67.

*2-(3-Hydroxyphenyl)-6-morpholino-[4,4'-bipyridine]-3-carbonitrile* (**9g**). The title compound was prepared from **7g**. After completion of the reaction, the mixture was diluted with water and the resulting precipitate was filtrated. The off-white solid was washed with water, dichloromethane, methanol, and dried, yield 45%, m.p. 290–292 °C dec. IR (KBr) 3063, 2860, 2197, 1586, 1509, 1269, 1252, 1114, 822 cm$^{-1}$. $^{1}$H-NMR (300 MHz, $(CD_3)_2SO$) δ 8.72 (s, 1-H), 8.75 (d, *J* = 6.1 Hz, 2-H), 8.65 (d, *J* = 6.1 Hz, 2-H), 7.35–7.19 (m, 3-H), 6.97 (s, 1-H), 6.91 (dt, *J* = 7.7/2.4 Hz, 1-H), 3.74 (br s, 4-H), 3.71 (br s, 4-H) ppm; $^{13}$C-NMR (75 MHz, $(CD_3)_2SO$) δ 161.7, 158.4, 157.3, 151.8, 150.0, 144.9, 139.5, 129.4, 123.5, 119.7, 118.5, 116.9, 115.8, 105.2, 92.1, 66.0, 44.6 ppm. MS (ESI) *m*/*z* 359 (M + H)$^{+}$. Anal. Calcd for $C_{21}H_{18}N_4O_2$: C, 70.38; H, 5.06; N, 15.63; Found: C, 70.12; H, 4.97; N, 15.54.

*2'-(3-Hydroxyphenyl)-6'-morpholino-[3,4'-bipyridine]-3'-carbonitrile* (**9h**). The title compound was prepared from **7h**. The crude material was purified by column chromatography using PE/EtOAc 5:5 as eluant, to give an off-white solid which was crystallized with EtOAc, yield 65%, m.p. 239–242 °C dec. IR (KBr) 3051, 2860, 2210, 1594, 1484, 1195, 1117, 710 cm$^{-1}$. $^{1}$H-NMR (300 MHz, $(CD_3)_2SO$) δ 9.68 (br s, 1-H), 8.84 (d, *J* = 1.8 Hz, 1-H), 8.72 (dd, *J* = 4.9/1.5 Hz, 1-H), 8.09 (dt, *J* = 8.6/1.5 Hz, 1-H), 7.59 (dd, *J* = 8.6/4.9 Hz, 1-H), 7.31–7.24 (m, 3-H), 7.02 (s, 1-H), 6.92 (dt, *J* = 7.3/2.5 Hz, 1-H), 3.77–3.70 (m, 8-H) ppm; $^{13}$C-NMR (75 MHz, $(CD_3)_2SO$) δ 161.5, 158.3, 157.2, 151.0, 150.2, 148.9, 139.5, 136.3, 133.2, 129.3, 123.4, 119.5, 118.6, 116.7, 115.7, 105.4, 92.7, 65.9, 44.5 ppm. MS (ESI) *m*/*z* 359 (M + H)$^{+}$. Anal. Calcd for $C_{21}H_{18}N_4O_2$: C, 70.38; H, 5.06; N, 15.63; Found: C, 70.38; H, 5.13; N, 15.67.

**Procedure for the Preparation of 2-(3-Hydroxyphenyl)-6-morpholino-4-methylnicotinonitrile (10) (Scheme S1).**

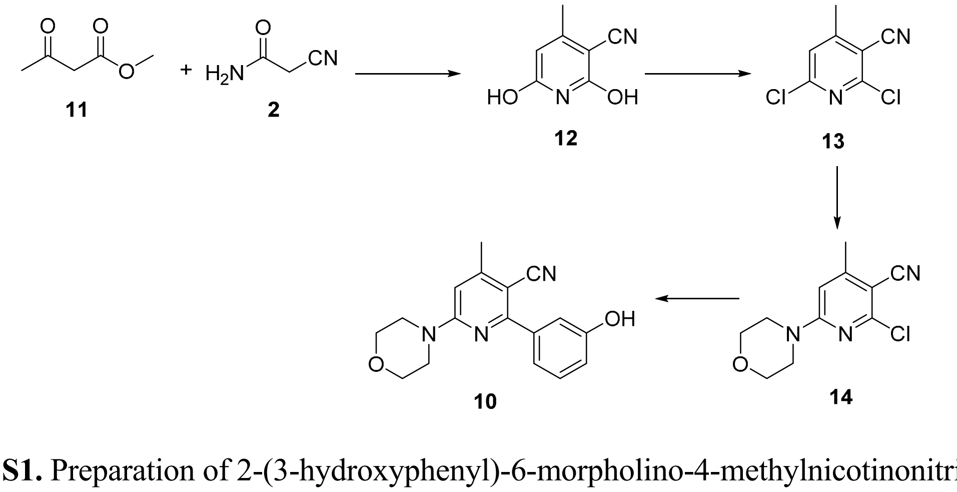


**Scheme S1.** Preparation of 2-(3-hydroxyphenyl)-6-morpholino-4-methylnicotinonitrile (**10**).

*2,6-Dihydroxy-4-methylnicotinonitrile* (**12**). The title compound was synthesized from commercially available methylacetoacetate (**11**) according to the literature procedure [9]. White solid, yield 90%, m.p. 298–300 °C. $^{1}$H-NMR (300 MHz, $(CD_3)_2SO$) δ 9.20 (br s, 1-H), 5.57 (s, 1-H), 2.17 (s, 1-H) ppm; $^{13}$C-NMR (75 MHz, $(CD_3)_2SO$) δ 164.3, 163.44, 162.8 119.5, 95.2, 91.4, 23.1 ppm.

*2,6-Dichloro-4-methylnicotinonitrile* (**13**). The title compound was prepared from 2,6-dihydroxy-4-methylnicotinonitrile (**12**) according to the general procedure C. Yellow solid, yield 95%, m.p.

109–110 °C, $^{1}$H-NMR (300 MHz, $CDCl_3$) δ 7.27 (s, 1-H), 2.57 (s, 3-H) ppm; $^{13}$C-NMR (75 MHz, $CDCl_3$) δ 159.1, 153.9, 153.2, 124.5, 113.7, 110.3, 21.2 ppm. The spectral data were consistent with those reported previously [10].

*2-Chloro-6-morpholino-4-methylnicotinonitrile* (**14**). The title compound was prepared from 2,6-dichloro-4-methylnicotinonitrile (**13**) according to the general procedure D. Off-white solid, yield 70%, m.p. 158–160 °C. $^{1}$H-NMR (300 MHz, $CDCl_3$) δ 6,33 (s, 1-H), 3.77–3.74 (m, 4-H), 3.63–3.61 (m, 4-H), 2.38 (s, 3-H) ppm; $^{13}$C-NMR (75 MHz, $CDCl_3$) δ 158.6, 153.8, 152.6, 115.9, 104.4, 97.8, 66.4, 44.8, 21.0 ppm.

*2-(3-Hydroxyphenyl)-6-morpholino-4-methylnicotinonitrile* (**10**). The title compound was prepared from 2-chloro-6-morpholino-4-methylnicotinonitrile (**14**) according to the general procedure E. Off-white solid, yield 72%, m.p. 195–197 °C. $^{1}$H-NMR (300 MHz, $(CD_3)_2SO$) δ 9.68 (s, 1-H), 7.29–7.18 (m, 3-H), 6.88 (brs, 2-H), 4.07 (brs, 4-H), 3.68 (brs, 4-H), 2.41 (s, 3-H) ppm; $^{13}$C-NMR (75 MHz, $(CD_3)_2SO$) δ 160.2, 158.4, 157.2, 152.1, 139.5, 129.3, 119.3, 118.3, 116.6, 115.4, 105.3, 94.7, 65.9, 44.4, 20.5 ppm. MS (ESI) *m/z* 296 $(M + H)^+$. Anal. Calcd for $C_{17}H_{17}N_3O_2$: C, 69.14; H, 5.80; N, 14.23; Found: C, 69.45; H, 6.11; N, 14.29.

**Additional Scheme, Table and Figures**

**Table S1.** The Chemgauss4 scores of compounds considered in the docking study.

| | PI3Kα | PI3Kγ |
|---|---|---|
| **9b** | −8.11 | −9.75 |
| **10** | −8.08 | −10.99 |

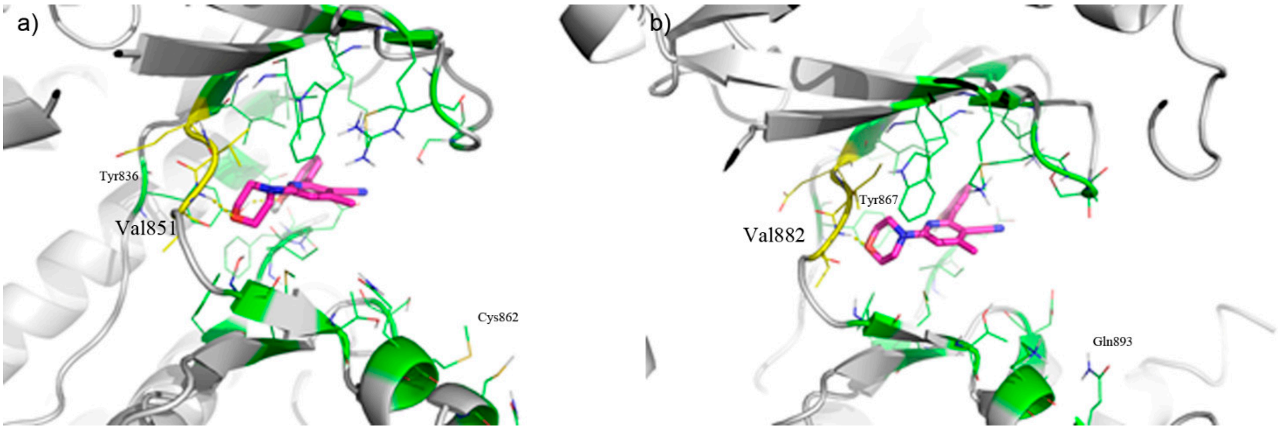


**Figure S1.** Predicted binding pose of compound **10** (purple sticks) in the PI3Kα (**a**) and PI3Kγ (**b**) binding sites (green sticks and white cartoon); amino acids of hinge region are shown as yellow sticks.

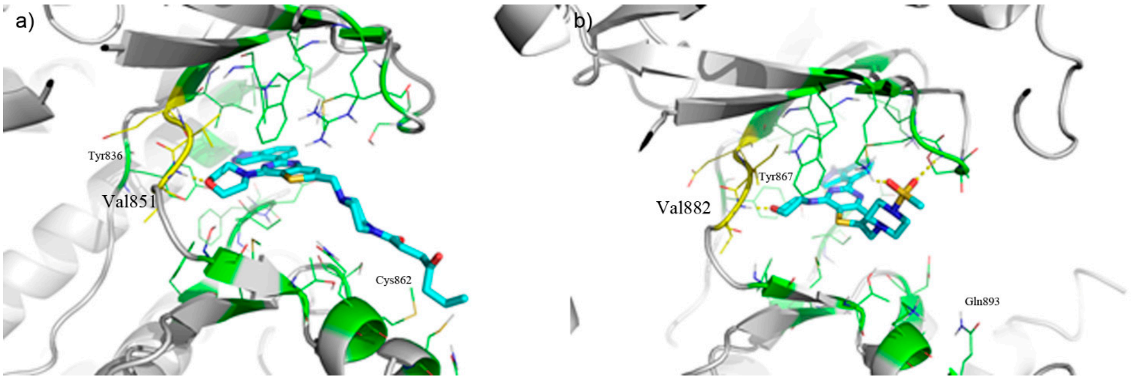


**Figure S2.** X-ray crystal structure used in the molecular docking study (green sticks and white cartoon), amino acids of hinge region are shown as yellow sticks and ligands as cyan sticks. (**a**) PI3Kα (PDB id: 3ZIM); (**b**) PI3Kγ (PDB id: 3DBS).

## References

1. Borowiecki, P.; Bretner, M. Studies on the chemoenzymatic synthesis of (*R*)- and (*S*)-methyl 3-aryl-3-hydroxypropionates: the influence of toluene-pretreatment of lipase preparations on enantioselective transesterifications. *Tetrahedron: Asymmetry* **2013**, *24*, 925–936.
2. Jimenez, J.M.; Knegtel, R.; Robinson, D.; Collier, P. Preparation of thiazoles, imidazoles, and pyrazoles useful as inhibitors of protein kinases for disease treatment. Patent WO2007117692 A2, 18 October 2007.
3. Pericas, A.; Shafir, A.; Vallribera A. Asymmetric synthesis of L-carbidopa based on a highly enantioselective α-amination. *Org. Lett.* **2013**, *15*, 1448–1451.
4. Zhou, B.; Hou, W.; Yang, Y.; Li, Y. Rhodium(III)-Catalyzed Amidation of Aryl Ketone *O*-Methyl Oximes with Isocyanates by C-H Activation: Convergent Synthesis of 3-Methyleneisoindolin-1-ones. *Chem. Eur. J.* **2013**, *19*, 4701–4706.
5. Eid, C.N., Jr.; Konopelski, J.P. Acylketene Acetals in Organic Synthesis. *Tetrahedron* **1991**, *47*, 975–992.
6. Zheng, H.J.; Chen, W.B.; Wu, Z.J.; Deng, J.G.; Lin, W.Q.; Yuan, W.C.; Zhang, X.M. Highly enantioselective synthesis of β-amino acid derivatives by the lewis base catalyzed hydrosilylation of β-enamino esters. *Chem. Eur. J.* **2008**, *14*, 9864–9867.
7. Pevet, I.; Brulé, C.; Tizot, A.; Gohier, A.; Cruzalegui, F.; Boutin, J.A.; Goldstein, S. Synthesis and pharmacological evaluation of thieno[2,3-*b*]pyridine derivatives as novel c-Src inhibitors. *Bioorg. Med. Chem.* **2011**, *19*, 2517–2528.
8. Alimmari, A.S.A.; Božić, B.D.; Marinković, A.D.; Mijin, D.Ž.; Ušćumlić, G.S. Solvent and Structural Effects on the UV–Vis Absorption Spectra of Some 4,6-Disubstituted-3-Cyano-2-Pyridones. *J. Solut. Chem.* **2012**, *41*, 1825–1835.
9. Panda, B. Guareschi cyclo-coupling of acetoacetate and cyanoacetamide; 3-cyano-2,6-dihydroxy-4-methylpyridine. In *ChemSpider SyntheticPages*; Royal Society of Chemistry: London, UK, 2008; doi:10.1039/SP267.
10. Katritzky, A.R.; Rachwal, S.; Smith, T.P.; Steel, P.J. Synthesis and reactivity of 2,6-diamino-4-methyl-3-pyridinecarbonitrile. *J. Heterocycl. Chem.* **1995**, *32*, 979–984.
